# Supplementary material for: Content-rich biological network constructed by mining PubMed abstracts
Source: BMC Bioinformatics. 2004 Oct 8;5:147. doi: 10.1186/1471-2105-5-147 (PMC528731; doi:10.1186/1471-2105-5-147)
Supplement: Additional File 2 — The original results of the above study (non-essential files are deleted to keep the file size under the limit set by BMC bioinformatics). [file 1471-2105-5-147-S2.bz2 › chilibotAdditionalFile2/dip20/35ID8530384E122/html/POU2F2_POU2AF1.html]

 


 **POU2F2** and **POU2AF1** 
  
Found 53 abstracts in PubMed, retrieved 20.  
 

 What does Google say? 
 PDF only 
| .edu only 

---

**Interactive relationship** (e.g. stimulation, inhibition, etc)

**Neutral relationship**- Here we report that overexpression of the Oct 2  [ **POU2F2** ]  transcription factor and octamer coactivator BOB  [ **POU2AF1** ] .1 OBF  [ **POU2AF1** ]  OCA B  [ **POU2AF1** ] , both of which are induced in T cells following T cell receptor signaling, synergistically up regulates CCR5 promoter activity via interaction with an octamer motif on the promoter.  Ref: 11071893 J Biol Chem, 2001
- Oct 2  [ **POU2F2** ]  and OBF 1  [ **POU2AF1** ]  also called OCA B  [ **POU2AF1** ]  or Bob 1  [ **POU2AF1** ]  are B cell specific transcription factors that bind to the conserved octamer site of immunoglobulin promoters, yet their role in immunoglobulin transcription has remained unclear.  Ref: 11135581 Nat Immunol, 2001
- These findings support the notion that Oct 2  [ **POU2F2** ]  regulates gene transcription by both OBF 1  [ **POU2AF1** ]  dependent and independent mechanisms.  Ref: 11937630 Nucleic Acids Res, 2002
- In addition, both factors are essential for germinal center formation, although OBF 1  [ **POU2AF1** ]  is more important than Oct 2  [ **POU2F2** ]  for IgG production after immunization.  Ref: 11135581 Nat Immunol, 2001
- The transcriptional coactivator BOB  [ **POU2AF1** ] .1 OBF  [ **POU2AF1** ] .1 confers B cell specificity on the transcription factors Oct1 and Oct2  [ **POU2F2** ]  at octamer site containing promoters.  Ref: 11238890 Mol Cell Biol, 2001
- The transcriptional coactivator OBF 1  [ **POU2AF1** ] , which interacts with Oct 1 and Oct 2  [ **POU2F2** ]  and the octamer site DNA, has been shown to be critical for development of a normal immune response and the formation of germinal centers in secondary lymphoid organs.  Ref: 11483517 EMBO J, 2001
- As Ig promoter binding factors, Oct 1 and Oct 2  [ **POU2F2** ]  each work together with a B lymphocyte specific cofactor OCA B  [ **POU2AF1** ]  OBF 1  [ **POU2AF1** ]  Bob 1  [ **POU2AF1** ]  that interacts with them through their POU DNA binding domains.  Ref: 11591767 J Immunol, 2001
- Our data indicate that the PORE type Oct1 or Oct2  [ **POU2F2** ]  dimer, rather than the monomer, is the primary target of the cofactor OBF1  [ **POU2AF1** ] .  Ref: 12727885 EMBO J, 2003
- Oct 2  [ **POU2F2** ]  regulates CD36 gene expression via a consensus octamer, which excludes the co activator OBF 1  [ **POU2AF1** ] .  Ref: 11937630 Nucleic Acids Res, 2002
- BOB  [ **POU2AF1** ] .1 OBF  [ **POU2AF1** ] .1 also called OCA B  [ **POU2AF1** ] , a B lymphocyte specific transcriptional coactivator, is recruited to octamer containing promoters by interacting with the Oct 1 or Oct 2  [ **POU2F2** ]  proteins.  Ref: 12207332 Eur J Immunol, 2002

**Non-interactive relationship** (e.g. studied together, co-existance, homology, etc.)

- We generated mice in which the lymphoid compartment was reconstituted with cells that lack both Oct 2  [ **POU2F2** ]  and OBF 1  [ **POU2AF1** ] .  Ref: 11135581 Nat Immunol, 2001
- Despite not being entirely cell specific, the strong nuclear expression of Oct2  [ **POU2F2** ]  and BOB  [ **POU2AF1** ] .1 OBF  [ **POU2AF1** ] .1 by germinal center derived lymphomas makes these antibodies a potentially useful tool in lymphoma diagnosis.  Ref: 11904338 Mod Pathol, 2002
- Our findings dismiss the concept that the different immunoglobulin expression in cHD and LPHD is due to disrupting mutations of immunoglobulin V genes in cHD but is most likely due to a down regulation of Oct2  [ **POU2F2** ]  and or BOB  [ **POU2AF1** ] .1 OBF  [ **POU2AF1** ] .1.  Ref: 11154228 Blood, 2001
- Oct 2  [ **POU2F2** ]  and Bob 1  [ **POU2AF1** ]  deficiency in Hodgkin and Reed Sternberg cells.  Ref: 11280769 Cancer Res, 2001
- it is closely related to the mixed cellularity CHL MCHL in respect of BSAP, BOB  [ **POU2AF1** ] .1, and Oct2  [ **POU2F2** ]  expression.  Ref: 12647220 Virchows Arch, 2003
- In conclusion, absence of functional Oct 2  [ **POU2F2** ]  and Bob 1  [ **POU2AF1** ]  cells represents a novel mechanism for immunoglobulin gene deregulation in H RS cells.  Ref: 11280769 Cancer Res, 2001
- Additionally, germinal center B cells showed stronger Oct2  [ **POU2F2** ]  and BOB  [ **POU2AF1** ] .1 OBF  [ **POU2AF1** ] .1 staining.  Ref: 11904338 Mod Pathol, 2002
- Thus, we have investigated the expression of Oct1, Oct2  [ **POU2F2** ] , and BOB  [ **POU2AF1** ] .1 OBF  [ **POU2AF1** ] .1 in human reactive lymphoid tissue and in a series of 140 Hodgkin and non Hodgkin s lymphomas.  Ref: 11904338 Mod Pathol, 2002
- The transcription factors BSAP, BOB  [ **POU2AF1** ] .1, Oct2  [ **POU2F2** ]  and MUM1 are sequentially expressed in normal B cell development.  Ref: 12647220 Virchows Arch, 2003
- Our results demonstrate an absence of Oct2  [ **POU2F2** ]  and or BOB  [ **POU2AF1** ] .1 OBF  [ **POU2AF1** ] .1 in cHD and a striking overexpression of Oct2  [ **POU2F2** ]  in LPHD.  Ref: 11154228 Blood, 2001
- On immunohistochemistry, the following phenotype was observed CD45, CD20, CD79a, PAX5 BSAP, BOB  [ **POU2AF1** ] .1, Oct 2  [ **POU2F2** ] , PU.1, Bcl 2, CD30, HLA DR, MAL protein, Bcl 6, MUM1 IRF4, CD10, CD21, CD15, CD138, CD68, and CD3.  Ref: 12507907 Am J Pathol, 2003
- This inactivity correlates with either reduced levels or even a complete lack of several B cell specific transcription factors required for their expression Oct 2  [ **POU2F2** ] , OBF 1  [ **POU2AF1** ] , PU.1, E47 E12, PAX 5 and EBF.  Ref: 12118370 Oncogene, 2002
- We recently demonstrated that defective immunoglobulin promoter transcription correlates with the down regulation of the B cell transcription factors Oct2  [ **POU2F2** ]  and BOB  [ **POU2AF1** ] .1 OBF  [ **POU2AF1** ] .1.  Ref: 11929801 Blood, 2002
- Analysis of octamer binding transcription factors Oct2  [ **POU2F2** ]  and Oct1 and their coactivator BOB  [ **POU2AF1** ] .1 OBF  [ **POU2AF1** ] .1 in lymphomas.  Ref: 11904338 Mod Pathol, 2002
- B cell development and immunoglobulin gene transcription in the absence of Oct 2  [ **POU2F2** ]  and OBF 1  [ **POU2AF1** ] .  Ref: 11135581 Nat Immunol, 2001
- Lack of Oct 2  [ **POU2F2** ]  and Bob 1  [ **POU2AF1** ]  points to a defect in transcription machinery in H RS cells and is associated with lack of immunoglobulin gene expression in these cells.  Ref: 11280769 Cancer Res, 2001
- Down regulation of BOB  [ **POU2AF1** ] .1 OBF  [ **POU2AF1** ] .1 and Oct2  [ **POU2F2** ]  in classical Hodgkin disease but not in lymphocyte predominant Hodgkin disease correlates with immunoglobulin transcription.  Ref: 11154228 Blood, 2001
- Furthermore, the reintroduction of BOB  [ **POU2AF1** ] .1 OBF  [ **POU2AF1** ] .1 and Oct2  [ **POU2F2** ]  into cultured HRS cells restored the activity of cotransduced immunoglobulin promoter constructs.  Ref: 11154228 Blood, 2001
- As immunoglobulin gene expression in B cells requires an interaction between octamer sites and the transactivating factors Oct 2  [ **POU2F2** ]  and Bob 1  [ **POU2AF1** ] , this study addresses the expression of the transcription factors Oct 2  [ **POU2F2** ]  and Bob 1  [ **POU2AF1** ]  in H RS cells.  Ref: 11280769 Cancer Res, 2001
- B lymphoma cell lines have been generated from mice with targeted mutations in the oct 2  [ **POU2F2** ] , OBF 1  [ **POU2AF1** ] , vav 1 and btk genes, as a model system that lacks these limitations and possesses additional potential for experimental manipulation.  Ref: 12848851 Immunol Cell Biol, 2003
- However, it differs from other aggressive B cell lymphomas in that it shows defective immunoglobulin production despite the expression of OCT 2  [ **POU2F2** ] , BOB  [ **POU2AF1** ] .1, and PU.1 transcription factors and the lack of IgV gene crippling mutations.  Ref: 12507907 Am J Pathol, 2003
- Transcripts of Bob 1  [ **POU2AF1** ] , a B cell specific co factor of Oct 2  [ **POU2F2** ] , could not be observed in these cell lines.  Ref: 11280769 Cancer Res, 2001
- Our data also demonstrate that CD36 is the first example of an Oct 2  [ **POU2F2** ]  dependent gene whose expression in B cells is independent of OBF 1  [ **POU2AF1** ] .  Ref: 11937630 Nucleic Acids Res, 2002
- The expression patterns of BCL6, CD138, Oct2  [ **POU2F2** ] , and BOB1  [ **POU2AF1** ]  in HRS cells of lrcHL showed differences to those of both cHL and lpHL.  Ref: 12670918 Cancer Res, 2003
- The impairment of immunoglobulin transcription with a down regulated synthesis of Oct2  [ **POU2F2** ]  and BOB  [ **POU2AF1** ] .1 OBF  [ **POU2AF1** ] .1 is the first established general recurrent defect found in HRS cells.  Ref: 11154228 Blood, 2001
- As immunoglobulin transcription is mainly regulated by the B cell transcription factors Oct2  [ **POU2F2** ]  and BOB  [ **POU2AF1** ] .1 OBF  [ **POU2AF1** ] .1, we analyzed 35 cases of LPHD, 32 cases of cHD, and 2 Hodgkin disease cell lines for the expression of these transcription factors and also in parallel for immunoglobulin expression.  Ref: 11154228 Blood, 2001
- Absence of Oct 2  [ **POU2F2** ]  and Bob 1  [ **POU2AF1** ]  protein expression in primary H RS cells was demonstrated by performing immunohistochemistry in 20 cases of classical Hodgkin s disease.  Ref: 11280769 Cancer Res, 2001
- Furthermore, in all cases either a complete lack or strong reduction in the expression of the Oct2  [ **POU2F2** ]  transcription factor and the BOB  [ **POU2AF1** ] .1 OBF  [ **POU2AF1** ] .1 coactivator were found.  Ref: 11342448 Blood, 2001
